# Instrumental balance assessment in Parkinson's disease and parkinsonism. A systematic review with critical appraisal of clinical applications and quality of reporting

**DOI:** 10.3389/fneur.2025.1528191

**Published:** 2025-01-29

**Authors:** Andrea Merlo, Lorenzo Cavazzuti, Maria Chiara Bò, Francesco Cavallieri, Maria Chiara Bassi, Benedetta Damiano, Sara Scaltriti, Valentina Fioravanti, Giulia Di Rauso, Giacomo Portaro, Franco Valzania, Mirco Lusuardi, Isabella Campanini

**Affiliations:** ^1^LAM - Motion Analysis Laboratory, Neuromotor and Rehabilitation Department, San Sebastiano Hospital, Azienda USL-IRCCS di Reggio Emilia, Reggio Emilia, Italy; ^2^Merlo Bioengineering, Parma, Italy; ^3^Neurology Unit, Neuromotor and Rehabilitation Department, Azienda USL-IRCCS di Reggio Emilia, Reggio Emilia, Italy; ^4^Medical Library, Azienda USL-IRCCS di Reggio Emilia, Reggio Emilia, Italy; ^5^Clinical and Experimental Medicine PhD Program, University of Modena and Reggio Emilia, Modena, Italy; ^6^Neuromotor and Rehabilitation Department, Azienda USL-IRCCS Reggio Emilia, Reggio Emilia, Italy

**Keywords:** Parkinson's disease, atypical parkinsonism, posturography, static balance, stabilometry

## Abstract

**Introduction:**

Patients with Parkinson's disease (pwPD) and atypical parkinsonism usually complain of impaired balance. Instrumental posturography is widely used to quantitatively assess static balance in pwPD but many posturographic parameters and protocols have been suggested. We aimed to appraise the use of static posturography in pwPD and atypical parkinsonism, and identify gaps hindering its translation into clinical routine.

**Methods:**

A systematic review on four databases. Study methodology, clinical aspects, assessment protocol, technical aspects, and transferability to clinical practice were critically appraised by a set of quality questions, scored on three levels (0, 0.5, 1). Total scores were used to assess overall studies' quality.

**Results:**

132 studies were included. The majority (105/132) was rated medium-quality. The domains “transferability to clinical practice” and “assessment protocol” received the lowest scores. The main flaw hindering portability to clinical settings was the lack of a stated rationale behind the choice of a specific protocol and the selection of the posturographic parameters. Missing reporting about the technical aspects employed to manage posturographic data and comprehensive instructions given to the patients further contributed to lower quality.

**Discussion:**

We provided recommendations for enhancing the clinical transferability of studies on static posturography to assess pwPD, including (1) discussing the rationale for choosing the assessment protocols and posturographic parameters, (2) detailing the inclusion criteria and select appropriate samples, and (3) reporting all the technical information to replicate the procedures and computations.

**Systematic review registration:**

International Prospective Register of Systematic Reviews (PROSPERO) on 6th February 2024 (ID CRD42024500777), https://www.crd.york.ac.uk/prospero/display_record.php?ID=CRD42024500777.

## 1 Introduction

Patients with Parkinson's disease (pwPD) or atypical parkinsonism [e.g., multiple system atrophy (MSA), progressive supranuclear palsy (PSP)] (1, 2) usually complain of impaired balance, reduced postural reflexes, freezing of gait, festination, and difficulty in reacting to an external perturbance (3). It has been estimated that pwPD experience two to three times more falls than healthy older adults (4–7). Such increased risk of falls and associated injuries progressively reduce patients' autonomy from caregivers and affect their quality of life (8).

The assessment of balance in pwPD is usually based on clinical scales (9–13), providing a qualitative evaluation of postural instability. Other dynamic tests have been developed to obtain quantitative information about patients' ability to maintain postural control (13–15). These assessments have been suggested to be more sensitive to advanced stages of PD, while they fail to detect differences in the early stages of the disease (16).

Instrumental posturography represents a valuable technique for assessing and monitoring pwPD, providing centimeter-accurate measurements, and therefore capable of early discriminating different patterns among patients (16). It has been suggested to monitor disease progression and understand the effectiveness of physiotherapeutic, pharmacological therapy, and invasive treatments such as Deep Brain Stimulation (DBS) (17–19). The wide availability of instrumentation for stabilometric assessment, with relatively low costs, has encouraged its dissemination in healthcare institutions, leading to increased scientific publications on the topic. A search on Pubmed, including the keywords “Parkinson's disease” and “posturography,” yielded nearly 500 scientific articles published in the past 24 years, with about 40 papers/year in recent years. More than 80% of these papers were published in Journals categorized as clinical, according to Scimago Journal and Country Rank (20). The remaining 20% have been published in engineering or multidisciplinary journals and focused on developing new indicators to be extracted from posturography data to describe patients' conditions. On the one hand, this helps to refine the research for the best outcome measures able to discriminate different patients; on the other hand, newly generated parameters must have a relevant clinical meaning for clinicians to interpret them accurately and integrate them effectively into their daily practice. Among the main limiting factors of instrumental posturography highlighted in the literature there are: the absence of a defined “normal pattern”, the lack of standardization of the protocols, and the large number of parameters that can be computed (21). This heterogeneous array of options in the literature, particularly in the absence of engineering expertise, can lead to uncertainty among physicians who need to select which parameter to use in their daily practice. Transferability to clinical practice should be the ultimate goal of biomedical research, aimed at improving clinicians' knowledge and, consequently, patient management from diagnosis to treatment.

We designed a critical appraisal to evaluate whether the reporting of studies on static posturography in pwPD is sufficiently comprehensive or requires targeted guidance to enhance quality. This approach aligns with similar reviews of other innovative methods, such as gait analysis combined with artificial intelligence (22). While previous works have clearly highlighted the heterogeneity of outcomes used in posturography research (16, 23, 24), none have offered practical guidance to establish a reference framework for improving the reporting of future studies.

This study aims to systematically review the literature on the use of static posturography to assess pwPD and atypical parkinsonism, appraise its clinical and technical aspects, and identify gaps hindering its translation into the clinical routine. The findings will be used to offer valuable recommendations aimed at enhancing external validity and replicability of future studies.

## 2 Methods

The current systematic review followed the Preferred Reporting Items for Systematic reviews and Meta-Analysis (PRISMA) 2020 guideline (25).

To increase this research's clarity, transparency, and reproducibility, the protocol was *a-priori* registered on the International Prospective Register of Systematic Reviews (PROSPERO) on 6th February 2024 (ID CRD42024500777).

### 2.1 Research question and strategies

The leading question for this investigation was: “How is posturography used in the assessment of static balance in pwPD and atypical parkinsonism?”.

A scientific librarian developed comprehensive and systematic searches. We did not use the PICO framework because neither the Intervention nor the Comparison components could be defined for most studies included in this review, which does not focus on evaluating the effectiveness of a specific intervention on a particular outcome. Therefore, the search strings were developed through an iterative process. The research was conducted in January 2024 within the following databases: Medline, Cinahl, Embase, and Scopus. Furthermore, a manual cross-reference search was performed on the reference lists of included articles. No time limits were set for publications to be included.

The complete search strategies can be consulted in Table 1.

**Table 1:** Search strategies, according to each database.

**Database**	**Strategy**
Medline (Pubmed)	(Parkinson^*^ OR canvas OR “multisystem^*^ atrophy” OR “progressive supranuclear palsy” OR corticobasal OR “GBA Parkinson” OR ”Parkinson Disease“[Mesh] OR ”Parkinsonian Disorders“[Mesh]) AND (balance OR stability OR equilibrium OR ”Postural Balance“[Mesh]) AND (posturograph^*^ OR ”force plate“ OR stabilo^*^ OR ”center of pressure“ OR ”centre of pressure“ OR COP)
Cinhal	('parkinson disease'/mj OR 'parkinsonism'/mj OR parkinson^*^:ab,ti OR canvas:ab,ti OR 'multisystem^*^ atrophy':ab,ti OR 'progressive supranuclear palsy':ab,ti OR corticobasal:ab,ti OR 'gba parkinson':ab,ti) AND (balance:ab,ti OR stability:ab,ti OR equilibrium:ab,ti OR 'body equilibrium'/exp) AND (posturograph^*^:ab,ti OR 'force plate':ab,ti OR stabilo^*^:ab,ti OR 'center of pressure':ab,ti OR 'centre of pressure':ab,ti OR cop:ab,ti)
Embase	[(MH ”Parkinson Disease“) OR (MH ”Parkinsonian Disorders“)] OR TI (Parkinson^*^ OR canvas OR “multisystem^*^ atrophy” OR “progressive supranuclear palsy” OR corticobasal OR “GBA Parkinson”) OR AB (Parkinson^*^ OR canvas OR “multisystem^*^ atrophy” OR “progressive supranuclear palsy” OR corticobasal OR “GBA Parkinson”) AND (MH ”Balance, Postural“) OR TI (balance OR stability OR equilibrium) OR AB (balance OR stability OR equilibrium) AND TI (posturograph^*^ OR ”force plate“ OR stabilo^*^ OR ”center of pressure“ OR ”centre of pressure“ OR COP) OR AB (posturograph^*^ OR ”force plate“ OR stabilo^*^ OR ”center of pressure“ OR ”centre of pressure“ OR COP)
Scopus	[TITLE-ABS-KEY (parkinson^*^ OR canvas OR ”multisystem^*^ atrophy“ OR ”progressive supranuclear palsy“ OR corticobasal OR ”GBA Parkinson“) AND TITLE-ABS-KEY (balance OR stability OR equilibrium) AND TITLE-ABS-KEY (posturograph^*^ OR ”force plate“ OR stabilo^*^ OR ”center of pressure“ OR ”centre of pressure“ OR cop)]

### 2.2 Eligibility criteria

The inclusion criteria were: (a) studies involving adult pwPD or other types of atypical parkinsonism; (b) studies investigating pwPD using static posturography, with any protocol that included barefoot bipedal upright stance acquired with eyes open on a firm surface, which will be from now on considered the “baseline condition” (along with the baseline condition, protocols could then involve other conditions, e.g., open/closed eyes, feet apart/together, firm/foam surface, cognitive tasks); (c) primary peer-reviewed studies (e.g., RCT, clinical study, observational study); (d) full text available in English.

The exclusion criteria were: (a) studies including only healthy individuals or patients with other pathologies; (b) studies not involving humans (i.e., modeling studies); (c) acquisition protocol based on dynamic posturography only (e.g., Sensory Organization Test, Functional Reach Test); (d) sessions acquired while wearing goggles or similar headsets; (e) studies in which patients were provided onscreen feedback of the position of their center of pressure to stabilize during the assessment; (f) patients holding their arms away from the body so that the center of pressure is shifted (e.g., Romberg position).

We were interested in all outcome measures derived from static posturography acquired in the baseline condition.

### 2.3 Study selection

After all databases were searched, reports were exported to EndNote20 (Clarivate Analytics, PA, USA), and duplicates were removed. The remaining studies were imported to Rayyan (26) online software. Two independent reviewers (LC and MCB) blindly screened their title, abstract, and full text. Discrepancies were discussed in a consensus meeting. If agreement could not be reached, a third researcher was called upon to solve any discrepancy (AM).

### 2.4 Data extraction and report

Two reviewers (MCB and LC) independently extracted the data. When the computation methods of the parameters were unclear, a third author (AM), a bioengineer with 20 years of experience in instrumental motion and posture analysis, was consulted. When necessary, the authors were contacted by email to obtain missing data from the reports, and information was added if they replied in 1 month.

The following information was summarized in a custom table: first author, year of publication, journal, aims of the study, sample size, patients' demographic and clinical characteristics, any treatment undergone by patients, protocol adopted for the posturographic assessment (including different conditions tested, number and duration of repetitions, and dosage of any drugs or brain stimulation), task conditions (including foot positioning, gaze fixation, and arm positions), technical details of the instrumentations and data processing (e.g., filters applied), any posturographic parameter derived from baseline posture. Parameters (means and standard deviations, median, or ranges) were retrieved from text, tables, or obtained from figures when necessary. Findings were also presented in a narrative synthesis, grouped by the five domains illustrated in the following paragraph.

### 2.5 Quality assessment

The three reviewers involved in the previous steps critically appraised the included studies. In case of doubts about the clinical characteristics of the sample, a neurologist with extensive expertise in pwPD was consulted.

To standardize the quality assessment procedure, a tailored set of quality questions was designed, tested on a random sample of 30 studies, and refined progressively until its final version, reported in Table 2.

**Table 2:** Tailored critical appraisal for the assessment of the included studies, addressing five domains that should always be described.

**Q**	**Domain**	**Question**
Q1	Study methodology	Are the objectives of the study clearly stated? 0 = completely not stated 0.5 = authors described/introduced what they did 1 = the aim is clearly stated at the end of the introduction
Q2	Transferability to clinical practice	Are the objectives linked to daily clinical applications? 0 = no clinical application introduced 0.5 = clinical applications are not stated but can be derived from the introduction 1 = clinical applications are explicitly stated
Q3	Study methodology	Is the study design stated and the manuscript written accordingly? 0 = study design is not stated and the paper is not structured according to any appropriated checklist 0.5 = study design is not explicitly stated but the paper is structured according to the specific checklist 1 = study design is made clear in the manuscript and the paper is written accordingly
Q4	Clinical aspects	Are the characteristics of the sample sufficiently described (inclusion and diagnostic criteria, age, severity of the disease, etc.)? 0 = no disease-related information is provided to characterize the sample 0.5 = general inclusion criteria or sample characteristics are provided (diagnostic criteria only, or clinical scales only) 1 = sample is described according to diagnostic criteria, precise inclusion criteria, and clinical scales specific for the pathology (only for DBS study: stimulation parameters reported)
Q5	Study methodology	Are the sample characteristics adequate to address the aim of the study in terms of disease severity (e.g., homogeneity or stratification in descriptive and intervention studies, values distributed between minimum and maximum possible values in correlation studies)? NA = not applicable (for case studies or case series) 0 = no disease-related information is provided 0.5 = descriptive/intervention study includes a heterogeneous sample without stratification; correlation study includes a homogeneous sample but this represents only a subgroup of the population 1 = descriptive/intervention study includes a homogeneous sample or stratifies it; correlation study includes a heterogeneous sample representing the entire population
Q6	Study methodology	Is the included sample size justified by power computations or other methods, if applicable, or was an a-posteriori power analysis included? NA = not applicable (for case studies or case series) 0 = sample size was based on data availability and no a-posteriori power analysis was included 0.5 = sample size was based on data availability but a-posteriori power analysis was included 1 = sample size was a-priori designed based on power analysis computations
Q7	Clinical aspects	Are the characteristics of the rehabilitation treatment sufficiently described, when delivered (type of intervention, duration of each session, session weekly frequency, timeline and follow-ups….)? NA = not applicable (for observational studies) 0 = intervention is only cited 0.5 = intervention is described with few details (e.g., weekly frequency and duration) 1 = characteristics of the intervention are completely described to allow reproducibility
Q8	Technical aspects	Are the data acquisition and preprocessing parameters that may affect the posturographic parameters clearly reported (e.g., sampling frequency, low pass cut-off frequency, mean value removal)? 0 = no information is provided 0.5 = partial information is provided or can be retrieved from cited references 1 = parameters are entirely reported
Q9	Assessment protocol	Is the acquisition protocol sufficiently detailed to permit replication (duration of repetitions, number of repetitions, compliant/soft surface material)? 0 = no information is provided 0.5 = partial information is provided or can be retrieved from cited references 1 = parameters are entirely reported
Q10	Assessment protocol	Are the task conditions sufficiently detailed to permit replication (foot positioning, upper limb position, gaze fixation, instructions to the patient)? 0 = no information is provided 0.5 = partial information is provided or can be retrieved from cited references 1 = parameters are entirely reported
Q11	Assessment protocol	Are the testing conditions clear (ON/OFF drug condition, ON/OFF brain stimulation)? NA = not applicable for studies including patients diagnosed with parkinsonisms 0 = no testing information is reported 0.5 = testing conditions are cited 1 = information is reported in terms of dosage (LEDD or usual dosage), time of the day the drug was administered (washout clearly stated in case of OFF condition), hours from DBS starting
Q12	Transferability to clinical practice	Is the rationale for the selection of the assessment protocol (e.g., eyes open/closed, firm/foam surface, dual cognitive task, other foot positions) clearly stated? 0 = the rationale behind the choice of the protocol is not explained or cannot be derived across the manuscript 0.5 = the rationale behind the choice of the protocol can be derived across the manuscript 1 = the rationale behind the choice of the protocol is clearly stated
Q13	Transferability to clinical practice	Did the authors provide a rationale or discuss a-posteriori the choice of the posturographic parameters computed? 0 = the rationale behind the choice of the parameters is not explained or cannot be derived across the manuscript 0.5 = the rationale behind the choice of the parameters can be derived across the manuscript 1 = the rationale behind the choice of the parameters is clearly stated
Q14	Technical aspects	Was the computation of the posturographic parameters clearly described (definition, formulas, or reference to a paper with formulas)? 0 = definitions, formulas, or references for parameter computation are not reported 0.5 = some definitions, formulas, or references for parameter computation are reported 1 = definitions, formulas or references are provided for all parameters
Q15	Technical aspects	Are the units of measurement for each parameter clearly stated? 0 = units of measurements are never stated or are wrong 0.5 = units of measurements are stated only for few parameters or are not consistent across the manuscript 1 = units of measurements are clearly stated for each parameter
Q16	Technical aspects	Are the numerical data consistent with current literature? NA = not applicable when the parameter was used by fewer than five research groups^a^ 0 = data exceeding by 3 median absolute deviation (MAD) the overall median value 0.5 = data exceeding by 2 MAD the overall median value 1 = data are consistent with the median and MAD derived from the total number of the studies
Q17	Study methodology	Are the limitations of the study discussed (control arm, sample size, power analysis, external validity, follow-up timeline, protocol feasibility)? 0 = limitations are not discussed 0.5 = only few limitations are reported with limited discussion 1 = limitations are properly addressed
Q18	Study methodology	Are the conclusions consistent with the aim of the study? 0 = conclusions do not align with the aim 0.5 = conclusions partially recall the aim of the study 1 = conclusions are in line with the declared aim
Q19	Transferability to clinical practice	Are the clinical utility and transferability of the posturographic assessment addressed and discussed in the study? 0 = no clinical utility or transferability are discussed 0.5 = clinical utility or transferability are no explicitly addressed but can be derived from the discussion 1 = authors made clear the clinical implications and transferability of their study

^a^variables used by at least five research groups: 95% ellipse area (mm^2^); 90% ellipse area (mm^2^); area as sum of the triangles (mm^2^); RMS/SD_AP (mm); RMS/SD_ML (mm); RMS/SD (mm); velocity_AP (mm/s); velocity_ML (mm/s); velocity (mm/s); COP length_AP (mm); COP length_ML (mm); COP length (mm); COP position_AP (mm); COP position_ML (mm); sway range_AP (mm); sway range_ML (mm); COP sway_AP (mm) - mean displacement with respect to the center of the force plate; COP sway_ML (mm) - mean displacement with respect to the center of the force plate; maximum displacement_AP (mm); maximum displacement_ML (mm); mean displacement_AP (mm); radius (mm).

The questions, listed according to the traditional structure of the paper, explore five domains:

1) Study methodology (Q1, Q3, Q5, Q6, Q17, Q18), including study design, research question, homogeneity or stratification according to disease severity, sample size or power analysis computation, and statistical analysis;
2) Clinical aspects (Q4, Q7), including the description of the sample, and description of the intervention—if any;
3) Assessment protocol (Q9, Q10, Q11), including information about balance assessment procedures and their replicability;
4) Technical aspects (Q8, Q14, Q15, Q16), including computational operations and engineering data management;
5) Transferability to clinical practice (Q2, Q12, Q13, Q19), including the paper's added value to the literature to improve clinical management of pwPD.

Each question was scored on a three-level basis: 1 for yes, 0.5 for limited details, and 0 for no. For some items, the score Not Applicable (NA) was added. The total score was calculated for each study to evaluate its overall quality. The total score of each question—and, consequently, of each category—was then expressed as a percentage of the maximum achievable score (i.e., considering only the assessable studies), not to penalize studies that received an NA score for some items.

Finally, studies were categorized into three groups: high-quality (total score exceeding 80%), medium-quality (total score ranging between 51 and 79%), and low-quality (total score below 50%) as in Samadi Kohnehshahri et al. (22). We also separately evaluated the quality of each category, determined by the ratio of question scores at each level (low, medium, high) and the total number of questions linked to that category.

### 2.6 Differences in critical appraisal scores due to the journal subject area

We also investigated whether studies published in strictly clinical or mixed (i.e., clinical and bioengineering, bioengineering only, neurophysiology) journals received different scores in the categories of critical appraisal. We labeled the journal as clinical or mixed based on the main “subject area and category” on the Scimago Journal and Country Rank portal (20). When journals were not indexed on Scimago or classified as “multidisciplinary” (e.g., PlosOne), classification was carried out by examining the journal's aims, the study itself, and the authors' affiliations. Critical appraisal analysis was then performed for these two subgroups.

## 3 Results

**Figure 1:**
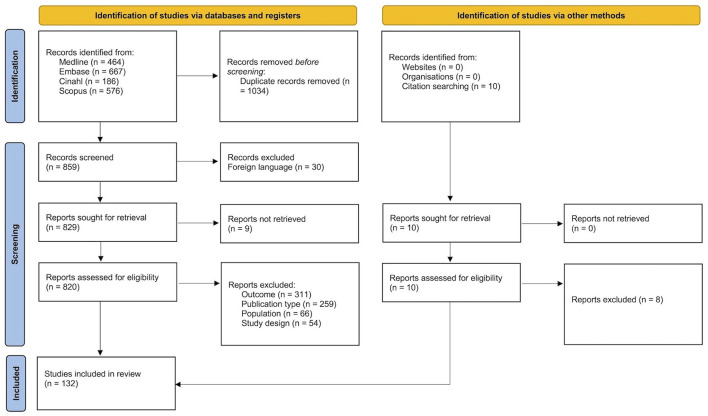
Prisma flowchart of the literature search on instrumental static balance assessment for patients with Parkinson's disease and atypical parkinsonism.

The original search identified 1,893 articles, which were turned into 859 after removing duplicates. These were screened for title and abstract according to the eligibility criteria. In this phase, the main reasons for exclusion were wrong outcomes or protocols employed (e.g., not involving the baseline condition, having patients performing dynamic balance tests) and wrong publication type (e.g., conference abstracts). Twenty-two studies required a third reviewer's intervention, and nine were included after consultation, leading to 130 eligible papers. In addition, two out of ten studies identified by hand searching were included, for a total of 132 studies included in the review (see the PRISMA Flowchart in Figure 1) (17–19, 27–155).

### 3.1 Data extraction

The comprehensive table containing all information extracted from the included studies can be found in Supplementary material 1.

Of the 132 studies, 115 focused on pwPD and 17 on atypical parkinsonism (MSA, PSP). Ninety-five were observational studies, and 37 were interventional studies. The former were generally cross-sectional studies aimed at describing the features of balance management of the included sample. The latter mainly evaluated a drug's or treatment's effectiveness on patients' postural ability.

Overall, 4,262 patients, aged 43–91, were assessed with static posturography in baseline condition.

### 3.2 Quality assessment

**Figure 2:**
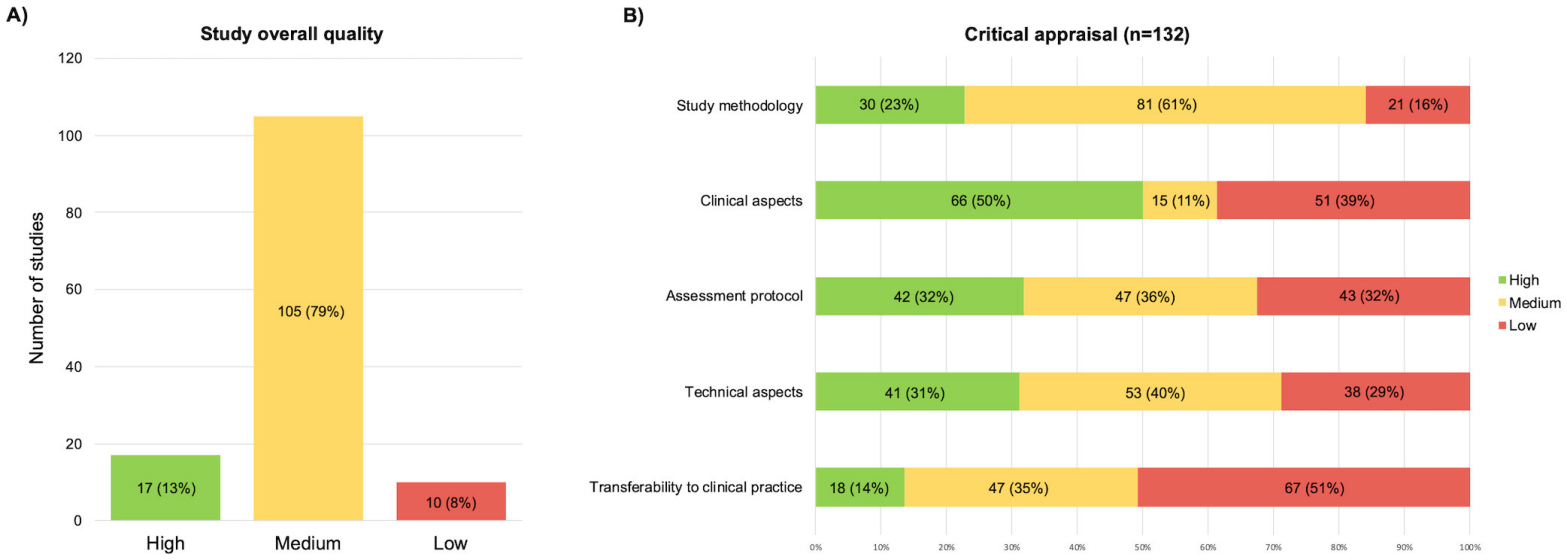
Graphic representation of the results of the critical appraisal, **(A)** overall scores; **(B)** scores split by the five domains of the critical appraisal.

The results obtained from the scores of each quality question are reported in Supplementary material 2. Of the 132 studies, 17 (13%) were rated high-quality, 105 (79%) medium-quality, and 10 (8%) low-quality (Figure 2A). The category “clinical aspects” received high-quality scores in half of the studies (see Figure 2B). The “technical aspects” and “study methodology” categories received mostly medium-quality ratings. Finally, the two categories with the lowest scores were “transferability to clinical practice” and “assessment protocol”.

Table 3 shows the results of each item from the five domains, detailing the average rating scores (see Supplementary material 2 for details of individual studies). The total score of each question was calculated as a percentage, excluding studies assessed as “NA” to avoid penalizing those questions. Consequently, some ratios have denominators lower than the total number of included studies (e.g., 74/128).

**Table 3:** Results of the critical appraisal for each item.

**Assessed domain**	**Results**
**Study methodology**	Mean rating: 69% (17% SD)
Q1—Study aims	115/132 (87%) authors specified the aims of their studies.
Q3—Study design	43/132 (33%) authors explicitly specified the study design.
Q5—Sample characteristics	- The included sample was appropriate in 74/128 studies (57.8%). - It was partially adequate in 45/128 studies (35.2%).
Q6—Sample size	Only 26 out of 127 (21%) studies computed a-priori the sample size or performed a-posteriori power analysis; specifically, 15/32 (47%) interventional and 11/95 (12%) observational studies did it.
Q17—Limitations	The limitation section was appropriately discussed in only 62/132 (47%) cases; barely mentioned in 40/132 (30%); and not even mentioned in as many as 30/132 (23%) cases.
Q18—Conclusions	119/132 (90%) authors wrote their conclusions consistently with the aims.
**Clinical aspects**	Mean rating: 75% (29% SD)
Q4—Sample description	68/132 (52%) of the studies accurately listed the eligibility criteria.
Q7—Intervention description	32/37 (87%) of the interventional studies described the intervention protocol in detail, allowing reproducibility.
**Assessment protocol**	Mean rating: 65% (23% SD)
Q9—Protocol description	89/132 (67%) and 32/132 (24%) studies accurately or sufficiently, respectively, described the acquisition protocol. The assessment protocol consisted of: - three balance trials in 49 (37%) studies; - one single trial in 47 (36%) studies, - two trials in 20 (15%) studies; - more trials (range 4–6) in 6 (4%) studies; - no information about the number of trials was reported in ten studies (8%) The duration of recordings ranged from 3 to 180 s, with 60/132 (45%) studies choosing 30 s as the duration of each session; 8/132 (6%) studies never stated the acquisition duration.
Q10—Task conditions	39/132 (30%) studies appropriately detailed the task conditions, while 70/132 (53%) authors provided only limited information: - 85/132 (64%) specified the instructions given to patients; - 67/132 (51%) specified the arm position; - 28/132 (21%) specified the position and type of visual target; - 60/132 (46%) explicitly stated they used the barefoot condition; - 50/132 (38%) provided numerical references for foot placement (e.g., intermalleolar distance, angle between feet); - 22/132 (17%) ensured standardized positioning by means of foot shapes or similar; - 23/132 (17%) gave general instructions to keep the feet at shoulder or hip width; - 6/132 (5%) let patients place their feet in undefined comfortable positions; - 29/132 (22%) specified nothing about foot positioning on platforms; - 7/132 (5%) reported that a quiet setting was ensured while assessing the patients. Beyond baseline conditions, the following conditions were also assessed among studies: - eyes closed on a firm surface (*n* = 83 studies), - eyes open on a soft surface (*n* = 14), - eyes closed on a soft surface (*n* = 14). - dual-task condition (*n* = 17 studies), usually asking patients to repeat sequences of numbers, count forward or backward, or say as many words as possible beginning with a given letter or belonging to a given category. - further protocols also included tandem and semi-tandem positions, unipedal standing, standing with feet together, or trunk or head inclined.
Q11—Drug and stimulation conditions	−52/126 (41%) studies adequately specified the drug or stimulation conditions while performing the balance assessment (i.e., the authors also specified the mean time since medication intake or the duration of the wash-out period). - Likewise, some of these studies also involved DBS and explained in the methods section the stimulation frequency used, the stimulus voltage, and the elapsed time between DBS onset and balance assessment.
**Technical aspects**	Mean rating: 68% (22% SD)
Q8—Data acquisition	−45/132 (34%) studies correctly described both the sampling frequency and the filtering process. - 53/132 (40%) papers reported only the sampling frequency, with no specification about the filters applied. Devices from about 20 different producers were used, including: - 3-axial for plates (*n* = 62), - monoaxial force plates - i.e., with vertical force sensors only (*n* = 46), - pressure mats (*n* = 18). - Nintendo Wii (*n* = 6) - Eleven studies did not report information on the device.
Q14—Parameter computation	−51/132 (39%) studies exhaustively described the computation process, with formulas, references, or clear definitions of the posturographic parameters. - 45/132 (34%) studies never provided any reference for parameter calculation. - The remaining 36 studies (27%) only partially described the formulas and computation methods.
Q15—Units of measurement	The units of measurements were uniformly made explicit in 122/132 (92%) studies, while five studies (4%) never reported any.
Q16—Parameter reliability	−82/109 (75%) studies reported values consistent with current literature. - 26/109 (24%) studies presented values even greater than three median absolute deviations away from the median for at least one parameter.
**Transferability to clinical practice**	Mean rating: 59% (19% SD)
Q2—Aims and clinical applications	The objectives of the studies were almost always explicitly related to clinical practice (*n* = 113/132, 86%).
Q12—Rationale behind the protocol	The rationale behind the protocol selection was never made explicit in 80/132 studies (61%); it could at most be derived from the text despite not being made explicit in 19/132 studies (14%).
Q13—Rationale behind the parameters	The rationale behind the choice of the parameter was explicitly stated in only 32/132 (24%) studies, while in the majority of the papers it was completely omitted (*n* = 68/132, 52%).
Q19—Discussion of clinical utility	Clinical utility was either made explicit (*n* = 76/132, 58%) or could be inferred from the discussions (*n* = 50/132, 38%).

SD, standard deviation; DBS, deep brain stimulation.

### 3.3 Differences in critical appraisal scores due to the journal subject area

Of the included studies, 102/132 were strictly clinical, and 30/132 were mixed studies.

Of the 102 clinical studies, 13 (13%), were rated high-quality, 81 (79%) medium-quality, and 8 (8%) low-quality. Of the 30 mixed studies, 4 (13%) were rated high-quality, 24 (80%) medium-quality, and 2 (7%) low-quality.

**Figure 3:**
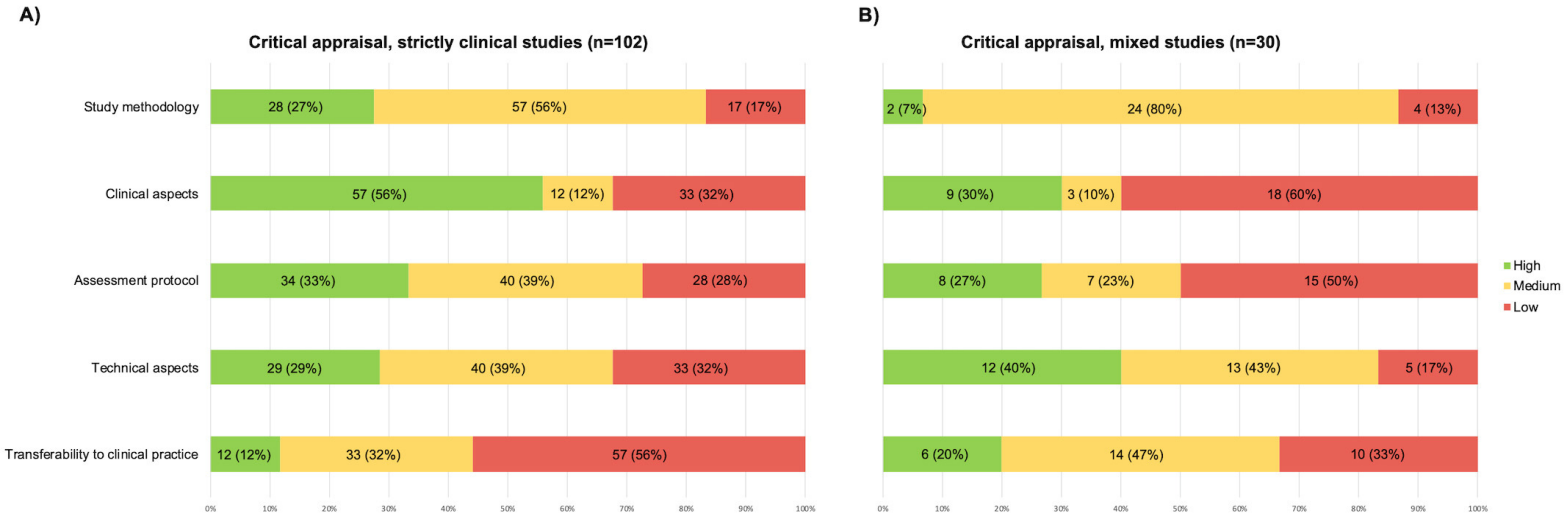
Graphic representation of the results of the critical appraisal, **(A)** papers published in clinical journals; **(B)** papers published in mixed journals.

Regarding clinical studies, the domains with the highest scores were “clinical aspects” and “assessment protocol” (see Figure 3A). The categories with the lowest scores were “transferability to clinical practice” and “technical aspects”. Thirteen clinical studies were cumulatively classified as high-quality. Only one study received high-quality ratings for all five domains (35).

Regarding mixed studies, the domain with the highest score was “technical aspects” (see Figure 3B). The categories with the lowest scores were “clinical aspects” and “assessment protocol”. Four mixed studies received high-quality global evaluation but no study received high-quality ratings for all five domains.

It is also worth mentioning that more than half of the studies received medium-quality scores for the domain “study methodology”.

## 4 Discussion

We conducted this systematic review to assess the use of static posturography in pwPD, analyzing which types of information are more detailed and how they may impact clinical transferability.

Based on the appraisal conducted, most of the included studies were rated as medium-quality, and only 17 studies were considered of high-quality (17, 32, 34–36, 47, 57, 63, 70, 112, 115, 117, 125, 126, 147, 148, 150).

The “”transferability to clinical practice” was the lowest-scoring domain at the critical appraisal, with only 14% of high-quality studies. From the results reported in Figure 2B and Table 3, this finding is attributable to three main factors. These are (1) The lack of discussion on the rationale adopted for choosing the posturographic protocol and computing the parameters used; (2) The conduction of the studies on limited, very heterogeneous, or insufficiently characterized samples from a clinical point of view; and (3) The lack of transparent reporting on the procedures for conducting the assessment and computing the parameters analyzed.

The interplay of these factors, identified through the critical appraisal items, prevented us from taking the next step in this review: summarizing the results as mean baseline values for pwPD at different stages of the pathology (e.g., H&Y score) or assessing the average variation in posturographic parameters following an intervention. A selection of studies with the highest appraisal scores was indicated above. The following paragraphs analyze these three points in the included studies, their impact on clinical transferability, and suggest possible solutions.

### 4.1 The rationale supporting the posturographic assessment of pwPD in the literature

Despite being rarely mentioned as a key point among the studies, three main clinical uses of the posturographic assessment in pwPD emerge from this review. These are: (1) to evaluate the effectiveness of rehabilitation treatments (37 clinical trials), (2) to monitor the course of the disease, and (3) to distinguish subgroups of patients (95 observational studies).

Only 16 studies have explicitly discussed how the assessment protocol and investigated posturographic parameters can be used in clinical routine (34–36, 38, 39, 42, 43, 50, 63, 72, 110, 115, 138, 147, 148, 150). Surprisingly, the lack of explanation of a clear rationale is particularly notable in studies published in purely clinical journals (see Figure 3A), which, on the contrary, are expected to be more accurate about this. This makes it difficult for readers to translate the literature into their daily clinical practice.

The reasons for this flaw may be manifold. It is possible that the authors took the rationale behind their choices for granted, as they are used to using these tools and dealing with these parameters every day. Also, the authors might have focused on the research-related aspects of their studies, so they did not discuss the clinical rationale and relevance of both their protocol and results. Next, several journals did not require to address this point explicitly. Concerning the choice of the posturographic parameters used in the studies included in this review, their selection may be due to—and limited by—the software of the commercial device used in the study. These, in fact, typically provide only the most used and standard outcomes, such as COP velocity and area, which may not be the most informative ones when assessing pwPD.

It is known that it takes about two decades to implement evidence into clinical practice (156). To bridge this time gap, the literature must focus on clinical relevance and transferability of findings. Many scientific journals require a short paragraph on clinical implications when submitting a manuscript. In line with this, future studies should discuss which additional information the posturographic test provides other than the clinical assessment, why it is essential to perform it in pwPD, and the possible contribution of the assessing protocol (task and parameters) to differential diagnosis or early recognition of symptoms or treatment effect assessment or patient status monitoring.

### 4.2 Characteristics of pwPD assessed by posturography in the literature

This systematic review included both pwPD and patients with atypical parkinsonism. To our knowledge, this is new in the literature, as this is the only review that included both populations. Despite being rarer, diseases such as PSP and MSA manifest clinical symptoms at onset that can be confused with PD and, therefore, deserve to be investigated as well and differentiated.

#### 4.2.1 The choice and description of the sample

A limited number of studies reported all the details about the sample, which is necessary to ensure the external validity of the results. Accurately describing sample characteristics allows readers to decide whether or not to compare with their reference patients. Clinical aspects were better described by the studies published in clinical journals (56% of high-quality studies), while the rate dropped to 30% for papers published in mixed journals. This is likely due to a greater sensitivity of clinical teams in reporting aspects that characterize the sample, compared to engineering groups.

Only 68/132 (52%) studies fully and accurately described the sample (see Q4). For pwPD, many studies reported diagnostic criteria and characterized patients through the Unified Parkinson's Disease Rating Scale (UPDRS) (157) and the Hoehn and Yahr staging (H&Y) (158) scales, as well as additional functional tests. For patients with atypical parkinsonism, a variety of specific scales can be used, depending on the disease, including the Unified Multiple System Atrophy Rating Scale, Progressive Supranuclear Palsy Rating Scale, and Scale for the Assessment and Rating of Ataxia (159–161). In progressive diseases such as PD, it might be relevant to report the time from the onset of the first symptoms or diagnosis, as well as the age and gender of the sample. In our review, the sample was considered “adequate” when it included patients with homogeneous characteristics in the case of intervention/descriptive studies and patients with heterogeneous characteristics in the case of correlation studies (see Q5). Among the included studies, 58% set the inclusion criteria consistently with the aim of the study. The adequacy of the sample allows the authors to identify specific findings for a homogeneous subgroup in terms of disease severity, supporting the translation of the results into clinical practice. Finally, the authors should specify whether the study was conducted solely for research purposes or if posturography acquisition was integrated as standard clinical practice.

When conducting a clinical study, the intervention should also be described. In our review, 86.5% of the studies detailed their protocols, including the type of intervention, frequency, and dosage, thus allowing reproducibility (see Q7). The TIDieR checklist was specifically developed in 2014 to improve the reporting quality for healthcare interventions and may further support authors during the writing of their Section 2 (162).

#### 4.2.2 The methodology used

The methodology domain has contributed to lowering the overall quality of the studies. The number of high-quality studies, already quite low when considering all studies (23%), drops to 7% when considering only studies published in mixed journals.

The main methodological limitation found in the included studies is the rare *a-priori* sample size calculation (see Q6). Only 24/127 (21%) of the studies calculated the sample size *a-priori*, along with two other studies (2%) that calculated statistical power *a-posteriori*. Focusing on interventional studies only, surprisingly, only half (47%) of them were conducted on a sample with an adequately calculated sample size. This is a relevant flaw because it can result in the absence of a statistically significant difference in the posturographic outcomes between the experimental and the control group in the presence of an actual difference (type-II error). The lack of this calculation limits the generalizability of their results and, again, their clinical transferability. Furthermore, it hinders the observation of potential differences between groups (in both observational and clinical studies), thereby impacting clinical decision-making (163).

When considering methodology, it is also appropriate to clarify the weaknesses of a study, which helps naïve readers not to overestimate the results and be aware of the limitations they might encounter if they intend to replicate the work in their daily activities. Only 62/132 studies comprehensively reported the limitations of their work (see Q17), and authors rarely referred to guidelines used during writing. Several reporting guidelines have been published since the late 1990s (e.g., CONSORT for experimental studies–1996) (164) and STROBE for observational studies in 2007 (165). More and more scientific journals require them to be completed during submission, proving that they are a handy tool for ensuring that the required methodological rigor has been met and that all the necessary information has been reported.

### 4.3 Issues in the reporting of protocol details and technical information in the literature

Two domains with similar percentages of high-quality studies are “assessment protocol” (32%) and “technical aspects” (31%). Many studies obtained fair scores at the critical appraisal as they correctly provided the necessary information. However, several technical and assessment-related aspects still deserve consideration.

#### 4.3.1 Assessment protocol

Studies that include posturographic examination should thoroughly describe how it is performed, and which protocol is adopted so as to allow replicability both in subsequent studies and in clinical practice. Key information to be reported is the number of sessions, their duration, the positioning of the feet and arms, the visual target provided, and the instructions given to the patient before the test.

##### 4.3.1.1 Number and duration of repetitions

In the current review, almost the same number of studies performed one or three repetitions, with durations ranging from 3 s to 3 min. Such short durations must never be included, as they are dramatically shorter than even the 5–10 s of recording recommended in some studies before collecting data for analysis (166, 167) and, therefore, provide no helpful information on COP characteristics. In the specific case (46) the authors reported the static balance assessment preliminary to gait analysis acquisitions.

The importance of averaging the parameters obtained in at least three successive trials to obtain values close to the subject's actual value is highlighted in the literature (168, 169). As the number of trials increases, the average value of the most commonly used posturographic parameters, such as COP area and velocity, stabilizes. At least 3–4 trials are necessary to stabilize posturographic parameters (167–170). In addition, the availability of multiple trials makes it possible to observe the variability of posturographic parameters between trials and provide further insight into the patient's condition.

The duration of the single test also affects the value of posturographic parameters. The literature has long investigated the best methods of standardizing the examination (166, 171–175), indicating durations between 30 and 90 s. Most (98/132, 74%) of the studies included in this review involved acquisitions with durations between 30 s (60/132, 45%) and 60 s (26/132, 20%).

The duration of 51.2 s—or multiples—observed in several studies (42, 53, 75, 101, 113, 129, 130, 146, 176) may seem strange to the reader and deserves a brief comment. This duration, adequate if not strenuous for the patient, does not derive from a physiological rationale. It depends on the technical characteristics of specific devices available in the 1980s and used for the definition of the first normative data through the work of the group of Gagey and Bizzo (172, 177). The availability of normative values encouraged, at first, the adoption of this duration in subsequent literature and by device manufacturers.

Furthermore, it should be kept in mind that for individuals such as pwPD, excessive duration could induce high levels of fatigue (178). Performing very long trials should be a conscious choice by researchers, possibly justified as done by Workman and colleagues (148). Therefore, evaluations for clinical uses must be based on a trade-off between several factors: the assessment duration, patient fatigue, and the need to obtain multiple trials to compare and mediate. It seems advisable to record 3–4 trials of 30–60s each.

##### 4.3.1.2 Task instructions

Task instructions are another critical factor for the clinical transferability of posturographic assessment. Of the included studies, only 39/132 (30%) specified the conditions under which the patients performed the balance tests, such as the directions given to the patients, the position of the arms and feet, and the visual target.

Instructions given to patients (e.g., “stand as still as possible” and “maintain a comfortable posture”) affect the reactions and the focus they will maintain during the test and, therefore, should be made explicit (179, 180). Similarly, the position of the arms (e.g., along the body, crossed) and the visual aim provided (e.g., “look straight ahead”, “look at a specific point at eye level”) could result in changes in the positioning and sway of the center of pressure (175). If possible, it would also be appropriate to specify that a quiet setting has been ensured throughout the performance of the test, mainly to avoid acoustic spatial orientation (166, 175), as done by some authors (65, 111–113, 116, 120, 148).

Unambiguous clarification of foot positioning is also essential (181). It is not sufficient to ask the patient to position themselves with their feet shoulder-width apart comfortably, nor to ask them to keep them open at shoulder or hip width (28/132 studies). This clearly does not allow for reproducibility. Other 18/132 studies let the patients position themselves at will but ensured position repeatability between trials by marking the initial position acquired. This is certainly a strength for internal validity, but it does not allow comparison of them with external literature and, again, limits the transferability of findings. Therefore, proper reporting of foot placement should always include numerical values for the distance between the feet and the angular opening between them. The literature suggests a distance of 3–5 cm at an angle of 30° (182) for patients with pathologies such that testing with feet together is not feasible and safe. Although it may seem obvious, researchers should always clarify whether the patient wears footwear or is barefoot during testing (only 60/132, 46%, studies among those included explicitly stated this), as the increased surface area—and sometimes, a slight ankle restraint provided by the footwear—, could affect the subject's stability (166).

The current review focused on the fair reporting of posturographic assessment under “baseline” conditions (i.e., eyes open, on a stable surface). Still, the same rigor must be maintained for any further testing. Specific tasks may reveal the patient's hidden postural issues, depending on individual strategies, as the dual-task condition (183). In this case, it is necessary to describe the required task (e.g., “count backward by three units starting from 100”, “list all the names you can think of referable to the animal category”) (18, 54, 67, 101, 103, 121, 140, 150, 153), without merely mentioning a generic cognitive task. Tasks of increasing difficulty require additional attention and may alter simultaneous balance control (184). Again, in the case of tests on unstable surfaces, the material (e.g., foam, gel), thickness (50, 82, 119, 121), and gel viscosity should be described (36, 118), as they change the multiple biomechanical variables in the foot, resulting in an alteration to the distribution of plantar pressures (185).

Finally, since these pwPD are almost always under a drug regimen, it is relevant to clarify the phase (ON/OFF) in which the examination is performed: it is not sufficient to state whether the patient was in the ON or OFF phase. If in OFF, the patient must have performed a wash-out of at least 12 h; if in ON, the peak of maximum action occurs about 1–2 h after the last intake (186). Only 52/126 (41%) studies included in the current review described it correctly. As with the rehabilitative intervention, it is equally important to specify the frequency, voltage of stimulus administration, and timing of stimulation.

#### 4.3.2 Technical aspects

In addition to information about the protocol, details on data analysis should always be reported in the manuscripts because of their impact on the results and to allow study reproducibility.

In particular, mean value removal, data filtering procedures, calculation of the parameters, and subsequent synthesis methods (e.g., calculation of the mean or median among the many trials, exclusion of the first and last seconds of each trial) should be reported. Using filters and techniques that deviate from traditional practice should be justified, as did Schmit et al. (132), who chose not to apply filters because they intended to characterize dynamic COP patterns in PD.

Studies published in mixed journals, i.e., engineering-contributing journals, better addressed technical aspects (40% high-quality studies vs. 28% high-quality studies published in clinical journals), probably reflecting a greater focus of bioengineering authors on this information. Overall, we observed a lack of standardization in data acquisition, filtering methods, and variable computation, when reported. Low pass filtering frequency, for example, was rarely reported despite its relevant effect on the computed parameters (see Supplementary material 1). This prevents meaningful comparisons between studies and hinders the possibility of synthesizing results.

##### 4.3.2.1 Instrumental set-up

1D- and 3D force plates were the most used devices in the studies included in this review. From a technical point of view, 1D force plates with three vertical load cells placed in the shape of an equilateral triangle or four vertical load cells placed in the shape of a rectangle are suited to obtain COP. 3D force plates, which are more expensive, were reasonably available in the centers and used for additional applications, such as gait analysis. Furthermore, despite the adequate technical characteristics of the Nintendo Wii (1D force plate), this device is not certified as a medical device. It cannot be used with patients for clinical assessments. Recent literature is investigating wearable sensors as low-cost tools capable of providing data on patients' balance that is easier to obtain (187). Under quasi-static conditions, the center-of-mass acceleration (measured by inertial sensors) is related to that of CoP (measured by force platforms) (188) and may serve as a cost-effective alternative to force platforms in specific contexts after validation specific to each pathology and impairment level.

Only forty-five out of 132 (34%) authors accurately reported the sampling frequencies and filters applied to the raw data, demonstrating knowledge of their impact on the data. PwPDs usually exhibit a tremor oscillating between 4 and 6 Hz (189). Hence, the sampling frequency must be high enough to observe information regarding COP oscillations.

##### 4.3.2.2 Computation of the posturographic parameters

Of the included studies, 51/132 (39%) correctly described the computed parameters using mathematical formulas, precise definitions, or citing other articles. Some terms are often used arbitrarily in the literature, which can lead to confusion and misinterpretation, with the high risk for the inexperienced reader of comparing parameters with the same name that hide different underlying calculations. The most striking example is that of the term “sway area/path area/total area,” used in as many as 20 studies without referring to any formula and therefore with potentially very different meanings (30, 40, 41, 45, 76, 78, 93, 97, 99, 103, 107, 111, 112, 130, 137, 144, 146, 151, 154, 176).

The urgency to create an unambiguous taxonomy emerges from this heterogeneous scenario. The historical reference to which most of the literature refers is Prieto et al. (190). Quijoux et al. have recently suggested a new classification of parameters into four categories, including positional (e.g., COP mean position), dynamic (e.g., COP velocity), frequency (e.g., spectral total power), and stochastic (e.g., sample entropy) features (23). Researchers can also rely on open libraries for complete analysis of posturographic data (23). A multidisciplinary approach, including bioengineers in the team, may be adopted to develop specific codes for calculating parameters that are useful to the clinician or to use the available open-source libraries. Moreover, a consensus conference with clinical and engineering experts may establish the best pwPD-specific parameters, providing helpful guidance for software development companies. Alongside the development of new parameters and the informed choice of them, research should be directed toward identifying their psychometric properties and threshold values, capable of distinguishing clinically significant changes, as has been done for spatiotemporal gait parameters for pwPD (191) or some posturographic parameters in healthy subjects (168, 170).

In the current review, five studies (19, 39, 60, 64, 176) never reported the units of measurement of posturographic parameters, and other five studies (18, 43, 55, 57, 155) only partially reported them. As many as 26/109 (24%) studies reported values much higher (>3 MAD) than those found in the literature. In some cases, this could be due to typos in the reported unit of measurement, but this confirms the need to promote clear standards and guidelines in the performance, analysis, and reporting of posturographic data.

To compare the posturographic data obtained during daily clinical practice with those published in the literature, it is mandatory that the clinical characteristics of the samples are comparable and that the acquisition protocol and technical handling of the data are identical. Studies that have accurately reported the necessary information and presented reliable data for comparison are: (17, 35, 115, 130) for pwPD with H&Y ≤ 2, (17, 101, 112, 115, 125, 130, 131) for pwPD with H&Y > 2, and (112) for atypical parkinsonism.

### 4.4 Limitations

This review has some limitations that need to be considered.

First, we included studies that reported balance assessment under the “baseline” condition. So, other protocols (e.g., dynamic posturography), which could provide additional relevant information and valuable hints for clinical practice, were not considered.

The search was conducted on four databases and by hand searching the references, but some relevant papers not indexed through the keywords entered in the search strings may have been missed.

Finally, the appraisal we used to assess the included studies on posturography in pwPD is new to the literature and is not a validated tool, as it was constructed *ad hoc* by the authors. Although we relied on similar examples to conceive the items (16, 23, 24) and develop the scoring system (22), followed an iterative process, and consulted with experts for its design, it is possible that some important aspects were not considered.

## 5 Conclusion

This systematic review assessed the use of static posturography for quantifying static balance in pwPD and atypical parkinsonism, focusing on the aspects that may hinder the transferability of research results to clinical practice. The main issues identified include a lack of rationale behind posturographic protocols and parameters and unclear sample inclusions. We highlighted several opportunities for enhancing the quality of studies on static posturography in assessing pwPD and atypical parkinsonism. In future studies, authors should: (1) discuss the rationale behind the choice of a specific assessment protocol and a posturographic parameter, (2) detail the inclusion criteria and select appropriate samples according to the aim of the study, and (3) report all the technical information necessary to replicate the procedures and computations. Addressing these areas can significantly improve scientific literature's external validity and clinical transferability to daily practice. This review provided valuable references for each of the five domains considered, supporting the rapid portability of findings to clinical settings.

## Data Availability

The original contributions presented in the study are included in the article/Supplementary material, further inquiries can be directed to the corresponding author.

## Supplementary material

Supplementary 1 Characteristics of the included studies, instructions given to the patients for the static balance test, and posturographic parameters computed in each study.

Supplementary 2 Results of the critical appraisal for each item and each of the included studies.
