# Supplementary material for: Instrumental balance assessment in Parkinson's disease and parkinsonism. A systematic review with critical appraisal of clinical applications and quality of reporting
Source: Front Neurol. 2025 Jan 29;16:1528191. doi: 10.3389/fneur.2025.1528191 (PMC11814473; doi:10.3389/fneur.2025.1528191)
Supplement: Supplementary 2 — Results of the critical appraisal for each item and each of the included studies. [file Data_Sheet_2.docx]

Supplementary Material

**Supplementary 2.** Results of the critical appraisal for each item and each of the included studies.

| **First author and year** | **Q1** | **Q2** | **Q3** | **Q4** | **Q5** | **Q6** | **Q7** | **Q8** | **Q9** | **Q10** | **Q11** | **Q12** | **Q13** | **Q14** | **Q15** | **Q16** | **Q17** | **Q18** | **Q19** | **Total score** | **Total score, %** |
| --- | --- | --- | --- | --- | --- | --- | --- | --- | --- | --- | --- | --- | --- | --- | --- | --- | --- | --- | --- | --- | --- |
| Ahn 2022 | 1 | 1 | 1 | 1 | 1 | 1 | 1 | 0 | 0 | 0 | NA | 0 | 0 | 0 | 1 | 1 | 0.5 | 1 | 0.5 | 11 | 61% |
| Ali 2021 | 1 | 1 | 0.5 | 1 | 0.5 | 0 | NA | 0.5 | 1 | 0 | NA | 0 | 0 | 0 | 1 | 0 | 1 | 1 | 0.5 | 9 | 53% |
| Apthorp 2020 | 1 | 1 | 0.5 | 0.5 | 0.5 | 0 | NA | 0 | 1 | 0.5 | 0.5 | 0 | 0 | 1 | 1 | NA | 0.5 | 1 | 1 | 10 | 59% |
| Armand 2009 | 0.5 | 1 | 1 | 1 | 1 | 0 | NA | 1 | 1 | 0 | 1 | 0 | 0 | 0.5 | 1 | 0 | 0 | 1 | 0.5 | 10.5 | 58% |
| Ayan 2023 | 1 | 1 | 1 | 1 | 1 | 0 | 0.5 | 0 | 0 | 0 | 0.5 | 0 | 0 | 0 | 1 | 1 | 0.5 | 1 | 0.5 | 10 | 53% |
| Bacha 2021 | 1 | 1 | 0.5 | 1 | 1 | 0 | 1 | 0.5 | 1 | 0.5 | 0 | 1 | 0.5 | 0.5 | 1 | 1 | 0 | 1 | 0.5 | 13 | 68% |
| Bao 2023 | 1 | 1 | 0.5 | 1 | 1 | 1 | NA | 1 | 1 | 1 | 0.5 | 0 | 1 | 1 | 1 | 0 | 1 | 1 | 1 | 15 | 83% |
| Baratto 2002 | 0.5 | 1 | 0 | 0 | 0 | 0 | NA | 1 | 1 | 1 | 0 | 0.5 | 1 | 1 | 1 | 1 | 0 | 0.5 | 1 | 10.5 | 58% |
| Barbieri 2016 | 1 | 1 | 0.5 | 1 | 1 | 0 | NA | 1 | 1 | 0.5 | 1 | 1 | 1 | 0.5 | 1 | NA | 1 | 1 | 1 | 14.5 | 85% |
| Barbieri 2019 | 1 | 1 | 0.5 | 1 | 1 | 1 | NA | 1 | 1 | 1 | 1 | 1 | 1 | 1 | 1 | 1 | 1 | 1 | 0.5 | 17 | 94% |
| Bekkers 2014 | 1 | 1 | 0.5 | 1 | 1 | 0 | NA | 0.5 | 1 | 0.5 | 1 | 1 | 1 | 0.5 | 1 | 1 | 1 | 0.5 | 1 | 14.5 | 81% |
| Bekkers 2018 | 1 | 1 | 0 | 1 | 1 | 0 | NA | 1 | 1 | 0.5 | 1 | 0 | 1 | 0.5 | 1 | 1 | 1 | 0.5 | 1 | 13.5 | 75% |
| Bello 2013 | 1 | 1 | 0.5 | 1 | 1 | 0 | 1 | 0 | 1 | 0 | 1 | 1 | 1 | 0 | 1 | NA | 0.5 | 0.5 | 0 | 11.5 | 64% |
| Beretta 2015 | 1 | 1 | 0.5 | 1 | 0.5 | 0 | NA | 1 | 1 | 1 | 1 | 1 | 1 | 0.5 | 0 | NA | 0.5 | 1 | 0 | 12 | 71% |
| Blaszczyk 2007 | 1 | 1 | 0.5 | 0.5 | 1 | 0 | NA | 0.5 | 0.5 | 0.5 | 1 | 0 | 1 | 1 | 1 | 0 | 0.5 | 1 | 1 | 12 | 67% |
| Blaszczyk 2011 | 1 | 0.5 | 0.5 | 0.5 | 1 | 0 | NA | 1 | 0.5 | 0.5 | 1 | 0 | 1 | 1 | 1 | 1 | 0.5 | 1 | 0.5 | 12.5 | 69% |
| Blaszczyk 2016 | 1 | 0.5 | 0.5 | 0.5 | 1 | 0 | NA | 1 | 0.5 | 0.5 | 0 | 1 | 1 | 1 | 1 | NA | 0.5 | 1 | 1 | 12 | 71% |
| Bonnet 2020 | 1 | 1 | 0.5 | 1 | 0.5 | 0 | NA | 0.5 | 1 | 0.5 | 1 | 1 | 1 | 0.5 | 0.5 | NA | 1 | 1 | 0 | 12 | 71% |
| Brachman 2021 | 1 | 1 | 0.5 | 0.5 | 0.5 | 0 | 1 | 0 | 0.5 | 0.5 | 1 | 0 | 0 | 0 | 1 | 1 | 1 | 0.5 | 0.5 | 10.5 | 55% |
| Buated 2016 | 1 | 1 | 0.5 | 1 | 0.5 | 0 | NA | 0 | 0.5 | 0.5 | 1 | 1 | 0 | 0 | 1 | 0.5 | 1 | 1 | 1 | 11.5 | 64% |
| Burleigh 1995 | 0.5 | 1 | 0 | 0.5 | 1 | 0 | NA | 0 | 0.5 | 0.5 | 1 | 0 | 0 | 0.5 | 1 | 0 | 0 | 0.5 | 0.5 | 7.5 | 42% |
| Cabeleira 2019 | 1 | 1 | 1 | 1 | 1 | 1 | NA | 1 | 1 | 0.5 | 1 | 0 | 0 | 1 | 1 | 1 | 1 | 1 | 1 | 15.5 | 86% |
| Cabrera-Martos 2020 | 1 | 1 | 1 | 1 | 0.5 | 1 | 1 | 0 | 0 | 0 | 1 | 0 | 0 | 0 | 1 | 1 | 1 | 1 | 0.5 | 12 | 63% |
| Cancela 2021 | 1 | 1 | 1 | 0.5 | 0.5 | 1 | 1 | 0.5 | 1 | 0.5 | 1 | 0 | 0 | 0.5 | 1 | NA | 1 | 1 | 0 | 12.5 | 69% |
| Cardoso da Silva 2017 | 1 | 1 | 1 | 0.5 | 1 | 0 | NA | 0.5 | 1 | 0.5 | 0 | 1 | 1 | 0.5 | 1 | 1 | 1 | 1 | 1 | 14 | 78% |
| Carpinella 2017 | 1 | 1 | 1 | 0.5 | 0.5 | 0 | 1 | 0.5 | 0.5 | 0 | 0.5 | 0 | 0 | 1 | 1 | 1 | 1 | 1 | 0.5 | 12 | 63% |
| Chastan 2008 | 1 | 1 | 0 | 0.5 | 1 | 0 | NA | 0.5 | 1 | 0.5 | 0 | 0.5 | 0 | 0.5 | 1 | 1 | 0 | 1 | 1 | 10.5 | 58% |
| Chen 2021 | 1 | 1 | 1 | 1 | 0.5 | 1 | 1 | 0 | 1 | 0.5 | 0 | 0 | 0 | 0.5 | 1 | 1 | 1 | 1 | 0.5 | 13 | 68% |
| Colnat-Coulbois 2004 | 1 | 1 | 0 | 0.5 | 0.5 | 0 | 1 | 0 | 1 | 1 | 0.5 | 0 | 0.5 | 0 | 0.5 | 1 | 0 | 1 | 0.5 | 10 | 53% |
| Colnat-Coulbois 2011 | 1 | 1 | 0.5 | 0.5 | 0.5 | 0 | NA | 0 | 1 | 0.5 | 0.5 | 0 | 0.5 | 0 | 1 | 1 | 0.5 | 1 | 0.5 | 10 | 56% |
| Correa 2019 | 1 | 1 | 1 | 1 | 1 | 1 | NA | 1 | 1 | 0.5 | 1 | 0 | 0 | 1 | 0.5 | 1 | 1 | 1 | 1 | 15 | 83% |
| D'Andrea Greve 2014 | 1 | 1 | 1 | 1 | 0.5 | 0 | NA | 1 | 0.5 | 1 | 1 | 0 | 0 | 0.5 | 1 | 0 | 0 | 0 | 1 | 10.5 | 58% |
| Dallaire 2024 | 1 | 1 | 1 | 1 | 0.5 | 1 | NA | 1 | 1 | 0.5 | 0.5 | 0 | 0 | 0.5 | 1 | 1 | 1 | 1 | 1 | 14 | 78% |
| Dana 2021 | 1 | 1 | 0.5 | 1 | 1 | 0 | NA | 1 | 1 | 0.5 | 0.5 | 0.5 | 0 | 0 | 0 | 1 | 0.5 | 1 | 1 | 11.5 | 64% |
| de Carvalho Costa 2022 | 0.5 | 0.5 | 0.5 | 1 | 1 | 0 | NA | 1 | 1 | 1 | 0.5 | 1 | 0.5 | 1 | 1 | 0 | 1 | 1 | 1 | 13.5 | 75% |
| De la Casa-Fages 2017 | 1 | 1 | 0.5 | 1 | 1 | 0 | 1 | 0.5 | 0.5 | 0.5 | 1 | 1 | 0.5 | 1 | 0.5 | NA | 0.5 | 1 | 1 | 13.5 | 75% |
| Degani 2020 | 1 | 1 | 0.5 | 0.5 | 0.5 | 0 | NA | 0.5 | 1 | 1 | 0 | 0.5 | 0 | 1 | 1 | 0 | 0 | 1 | 1 | 10.5 | 58% |
| Donà 2016 | 1 | 1 | 1 | 1 | 0.5 | 1 | NA | 1 | 0.5 | 0.5 | 0.5 | 1 | 1 | 1 | 1 | 1 | 1 | 1 | 1 | 16 | 89% |
| Ehgoetz Martens 2016 | 1 | 1 | 0.5 | 0.5 | 0.5 | 0 | 0 | 0 | 0 | 0.5 | 0.5 | 1 | 0 | 0 | 0 | NA | 1 | 1 | 1 | 8.5 | 47% |
| Espinoza-Valdes 2021 | 1 | 1 | 0.5 | 1 | 1 | 0 | NA | 0.5 | 1 | 1 | 0 | 0 | 1 | 1 | 1 | 1 | 1 | 1 | 1 | 14 | 78% |
| Fadil 2023 | 1 | 1 | 0.5 | 0.5 | 1 | 0 | NA | 0.5 | 0 | 0 | 0 | 0 | 0 | 0 | 1 | 1 | 1 | 1 | 0.5 | 9 | 50% |
| Fernandes 2015 | 1 | 1 | 1 | 0.5 | 1 | 0 | NA | 0.5 | 1 | 0.5 | 0.5 | 0 | 0.5 | 0.5 | 1 | 1 | 1 | 1 | 0.5 | 12.5 | 69% |
| Ferreira-Barbosa 2015 | 1 | 1 | 0.5 | 1 | 0.5 | 0 | NA | 0.5 | 1 | 0.5 | 0.5 | 0.5 | 1 | 0.5 | 1 | 1 | 0.5 | 0.5 | 1 | 12.5 | 69% |
| Ferreira-Peruzzo 2023 | 1 | 1 | 0.5 | 1 | 0.5 | 0 | NA | 0.5 | 0 | 1 | 1 | 0.5 | 0.5 | 0 | 1 | 1 | 1 | 1 | 1 | 12.5 | 69% |
| Franzoni 2018 | 1 | 1 | 1 | 1 | 0.5 | 1 | 1 | 1 | 1 | 0.5 | 0.5 | 0 | 0 | 1 | 1 | 1 | 1 | 1 | 1 | 15.5 | 82% |
| Geroin 2015 | 1 | 1 | 1 | 1 | 0.5 | 0 | NA | 0 | 1 | 1 | 0 | 1 | 1 | 0.5 | 1 | 1 | 0.5 | 1 | 0.5 | 13 | 72% |
| Geroin 2019 | 1 | 1 | 1 | 1 | 1 | 0 | NA | 0 | 1 | 1 | 0 | 1 | 0.5 | 0 | 1 | 1 | 0.5 | 1 | 0.5 | 12.5 | 69% |
| Gervasoni 2015 | 1 | 1 | 0.5 | 0.5 | 0.5 | 0 | NA | 0.5 | 0.5 | 0.5 | 0 | 0.5 | 0.5 | 0.5 | 1 | 1 | 1 | 1 | 0.5 | 11 | 61% |
| Geurts 2010 | 1 | 1 | 0.5 | 0.5 | 0.5 | 0 | NA | 1 | 1 | 1 | 1 | 0 | 0.5 | 1 | 1 | NA | 0.5 | 1 | 1 | 12.5 | 74% |
| Guehl 2006 | 1 | 1 | 0.5 | 0.5 | 1 | 0 | 1 | 0.5 | 0.5 | 0.5 | 1 | 0 | 0 | 0.5 | 1 | 0 | 0.5 | 0.5 | 1 | 11 | 58% |
| Gulcan 2022 | 1 | 1 | 0.5 | 0.5 | 1 | 1 | 0.5 | 0 | 0 | 0 | 0.5 | 0 | 0 | 0 | 1 | 1 | 0.5 | 1 | 0.5 | 10 | 53% |
| Halmi 2019 | 1 | 1 | 0.5 | 0.5 | 1 | 0 | NA | 0.5 | 0.5 | 0 | 1 | 0 | 0 | 0 | 1 | 1 | 0.5 | 1 | 1 | 10.5 | 58% |
| Han 2013 | 1 | 1 | 0.5 | 0 | 0 | 0 | NA | 0.5 | 0 | 0 | 0 | 0 | 0 | 0 | 1 | 1 | 0 | 1 | 1 | 7 | 39% |
| Hasegawa 2021 | 1 | 1 | 0.5 | 0.5 | 1 | 0 | NA | 0.5 | 0.5 | 0.5 | 0.5 | 0 | 0 | 0 | 1 | NA | 1 | 1 | 0.5 | 9.5 | 56% |
| High 2018 | 1 | 1 | 0.5 | 0.5 | 0.5 | 0 | NA | 0.5 | 1 | 0.5 | 0.5 | 1 | 0 | 1 | 1 | 0 | 0.5 | 1 | 0.5 | 11 | 61% |
| Iwai 2021 | 1 | 1 | 1 | 0.5 | NA | NA | 1 | 1 | 1 | 0.5 | 0.5 | 0.5 | 0.5 | 0 | 1 | 0 | 0.5 | 1 | 0.5 | 11.5 | 68% |
| Jazaeri 2018 | 1 | 1 | 0.5 | 0.5 | 1 | 0 | NA | 0.5 | 1 | 0.5 | 1 | 0.5 | 1 | 0 | 1 | 0 | 1 | 1 | 0 | 11.5 | 64% |
| Jehu 2018 | 1 | 1 | 0.5 | 0.5 | 1 | 0 | NA | 1 | 1 | 0.5 | 0.5 | 0 | 0.5 | 0 | 1 | 1 | 1 | 1 | 1 | 12.5 | 69% |
| Johnson 2013 | 0.5 | 1 | 0.5 | 1 | 1 | 0 | NA | 0 | 1 | 0.5 | 0.5 | 0.5 | 0.5 | 0.5 | 1 | 1 | 0 | 1 | 1 | 11.5 | 64% |
| Johnson 2015 | 0.5 | 1 | 0.5 | 0.5 | 0.5 | 0 | 0.5 | 0 | 1 | 1 | 0.5 | 0 | 0.5 | 0.5 | 1 | 1 | 1 | 1 | 1 | 12 | 63% |
| Kamieniarz 2021 | 1 | 1 | 1 | 0.5 | 1 | 0 | NA | 0.5 | 1 | 0.5 | 0.5 | 0 | 1 | 1 | 1 | 1 | 1 | 0.5 | 1 | 13.5 | 75% |
| Karimi 2015 | 1 | 0.5 | 0.5 | 0 | 0 | 0 | NA | 1 | 0.5 | 0 | 0 | 0 | 0.5 | 1 | 1 | 0 | 1 | 1 | 1 | 9 | 50% |
| Kim 2016 | 1 | 1 | 0.5 | 0.5 | 0.5 | 0 | NA | 1 | 0.5 | 0.5 | 0 | 0 | 0.5 | 1 | 1 | 1 | 0 | 1 | 1 | 11 | 61% |
| Kim 2017 | 1 | 1 | 0.5 | 0.5 | 0.5 | 0 | 1 | 0.5 | 0.5 | 0.5 | 0 | 0.5 | 0.5 | 0 | 1 | 1 | 1 | 1 | 0.5 | 11.5 | 61% |
| Korkusuz 2023 | 1 | 1 | 0.5 | 1 | 0.5 | 1 | NA | 0 | 0.5 | 0.5 | 0.5 | 0 | 0 | 0.5 | 1 | 1 | 0.5 | 1 | 0.5 | 11 | 61% |
| Kudrevatykh 2020 | 1 | 1 | 0.5 | 1 | 1 | 0 | NA | 0.5 | 0.5 | 0.5 | 0 | 0 | 0 | 0 | 1 | NA | 0 | 1 | 1 | 9 | 53% |
| Kwon 2020 | 1 | 1 | 0.5 | 0.5 | 0 | 0 | NA | 1 | 1 | 0.5 | 0 | 0 | 0 | 1 | 1 | 1 | 0 | 1 | 1 | 10.5 | 58% |
| Kwon 2023 | 1 | 1 | 0.5 | 1 | 0.5 | 0 | NA | 0.5 | 1 | 1 | 0.5 | 0 | 0 | 1 | 1 | 1 | 0.5 | 1 | 1 | 12.5 | 69% |
| Kwon 2023 | 1 | 1 | 0.5 | 0.5 | 1 | 0 | NA | 1 | 0.5 | 0.5 | 0.5 | 0 | 0 | 0.5 | 1 | 1 | 0.5 | 1 | 1 | 11.5 | 64% |
| Lahr 2015 | 1 | 1 | 0.5 | 0.5 | 0.5 | 0 | NA | 0 | 0.5 | 0.5 | 0.5 | 0 | 0 | 1 | 1 | 1 | 0.5 | 1 | 1 | 10.5 | 58% |
| Lauretani 2016 | 1 | 0.5 | 1 | 1 | NA | NA | 1 | 0 | 0.5 | 0.5 | 0 | 0 | 0 | 0 | 1 | 0 | 0 | 1 | 1 | 8.5 | 50% |
| Lazarotto 2020 | 1 | 1 | 1 | 0.5 | 0.5 | 0 | NA | 0.5 | 1 | 0.5 | 0.5 | 0 | 0 | 0 | 1 | 1 | 0.5 | 1 | 1 | 11 | 61% |
| Lee 2016 | 1 | 1 | 0.5 | 0.5 | 1 | 0 | NA | 0.5 | 0.5 | 0.5 | 0.5 | 0 | 0.5 | 1 | 1 | 1 | 1 | 1 | 1 | 12.5 | 69% |
| Li 2020 | 1 | 0.5 | 0.5 | 0.5 | 0.5 | 0 | NA | 1 | 1 | 1 | 0 | 0.5 | 0.5 | 1 | 1 | 0 | 0.5 | 1 | 0 | 10.5 | 58% |
| Mancini 2011 | 1 | 1 | 0.5 | 0.5 | 0.5 | 0 | NA | 1 | 0.5 | 0.5 | 0 | 0 | 1 | 1 | 1 | 1 | 0.5 | 1 | 1 | 12 | 67% |
| Marchese 2003 | 1 | 0.5 | 0.5 | 1 | 1 | 0 | NA | 0.5 | 1 | 1 | 1 | 1 | 0 | 1 | 1 | 1 | 1 | 0.5 | 0.5 | 13.5 | 75% |
| Mirahmadi 2018 | 1 | 1 | 0.5 | 1 | 0.5 | 0 | NA | 1 | 1 | 0.5 | 0.5 | 0 | 1 | 1 | 1 | 0 | 0.5 | 1 | 1 | 12.5 | 69% |
| Morenilla 2020 | 1 | 1 | 0.5 | 0.5 | 0.5 | 0 | NA | 0.5 | 1 | 1 | 0.5 | 0.5 | 0 | 0.5 | 1 | 1 | 1 | 1 | 1 | 12.5 | 69% |
| Nantel 2012 | 1 | 1 | 0.5 | 1 | 1 | 0 | NA | 1 | 0.5 | 0.5 | 1 | 0 | 0 | 1 | 1 | 1 | 0 | 1 | 1 | 12.5 | 69% |
| Nantel 2014 | 1 | 1 | 0.5 | 0.5 | 0.5 | 0 | NA | 1 | 0.5 | 0.5 | 1 | 0 | 0 | 1 | 1 | 1 | 0 | 1 | 1 | 11.5 | 64% |
| Nardone 2006 | 0.5 | 0.5 | 0.5 | 0.5 | 1 | 0 | NA | 0 | 1 | 0.5 | 0.5 | 1 | 0 | 0 | 0 | NA | 0 | 1 | 1 | 8 | 47% |
| Nikaido 2018 | 1 | 0.5 | 0.5 | 1 | 1 | 0 | NA | 0.5 | 0.5 | 1 | 0.5 | 0.5 | 0.5 | 1 | 1 | NA | 1 | 1 | 1 | 12.5 | 74% |
| Nocera 2010 | 1 | 1 | 0.5 | 1 | 0.5 | 0 | NA | 0 | 1 | 0.5 | 0.5 | 0 | 0.5 | 0.5 | 1 | NA | 1 | 0.5 | 1 | 10.5 | 62% |
| Oz 2023 | 1 | 1 | 0.5 | 0 | 0 | 0 | NA | 0 | 1 | 0.5 | 0.5 | 0 | 0 | 0.5 | 1 | 1 | 0.5 | 1 | 0.5 | 9 | 50% |
| Padovan 2023 | 1 | 1 | 0.5 | 1 | 1 | 0 | NA | 0.5 | 0 | 0.5 | NA | 1 | 1 | 0.5 | 1 | 1 | 1 | 1 | 1 | 13 | 76% |
| Panyakaew 2015 | 1 | 1 | 0.5 | 0.5 | 0.5 | 0 | NA | 0.5 | 1 | 1 | 1 | 1 | 0.5 | 0.5 | 1 | 1 | 0.5 | 1 | 1 | 13.5 | 75% |
| Panyakaew 2019 | 1 | 1 | 0.5 | 1 | 1 | 1 | NA | 0.5 | 1 | 1 | 1 | 1 | 0.5 | 0.5 | 1 | 1 | 1 | 1 | 1 | 16 | 89% |
| Paolucci 2018 | 1 | 1 | 1 | 0.5 | 0.5 | 0 | NA | 0 | 1 | 1 | 0.5 | 0.5 | 0.5 | 0.5 | 1 | 1 | 0.5 | 1 | 1 | 12.5 | 69% |
| Park 2015 | 1 | 1 | 0.5 | 0 | 0 | 0 | NA | 1 | 1 | 0.5 | 0 | 0 | 0.5 | 1 | 1 | 1 | 0.5 | 1 | 1 | 11 | 61% |
| Pelykh 2015 | 1 | 1 | 0.5 | 1 | 1 | 0 | NA | 1 | 1 | 1 | 0.5 | 1 | 1 | 1 | 1 | 1 | 1 | 1 | 1 | 16 | 89% |
| Perera 2018 | 0.5 | 1 | 0.5 | 1 | 1 | 0 | NA | 0.5 | 1 | 0.5 | 1 | 0 | 0 | 1 | 1 | 1 | 1 | 0.5 | 0.5 | 12 | 67% |
| Piras 2022 | 1 | 1 | 0.5 | 0.5 | 1 | 1 | 1 | 1 | 1 | 1 | 1 | 1 | 0 | 0 | 1 | 1 | 1 | 1 | 1 | 16 | 84% |
| Qiu 2013 | 1 | 1 | 0.5 | 1 | 1 | 1 | 1 | 0.5 | 0.5 | 1 | 0 | 0 | 0 | 0 | 1 | 1 | 0 | 1 | 0.5 | 12 | 63% |
| Raethjen 2020 | 0.5 | 1 | 0.5 | 1 | 1 | 0 | 1 | 0.5 | 1 | 0.5 | 0 | 0 | 0 | 1 | 0 | NA | 0.5 | 1 | 1 | 10.5 | 58% |
| Rahmati 2019 | 1 | 1 | 0.5 | 1 | 0.5 | 0 | 1 | 1 | 0.5 | 0 | 1 | 0 | 1 | 1 | 1 | 1 | 1 | 1 | 1 | 14.5 | 76% |
| Ramos Pereira 2021 | 1 | 1 | 1 | 0.5 | 0.5 | 0 | 1 | 0 | 0 | 0 | 0 | 0 | 0 | 0 | 1 | NA | 0 | 1 | 0.5 | 7.5 | 42% |
| Raymakers 2005 | 1 | 0.5 | 0.5 | 0 | 0 | 0.5 | NA | 0.5 | 0.5 | 1 | 0 | 0 | 1 | 1 | 1 | 1 | 0 | 1 | 0.5 | 10 | 56% |
| Rezvanian 2018 | 1 | 1 | 0.5 | 0.5 | 1 | 0 | NA | 1 | 1 | 0.5 | 1 | 0 | 1 | 1 | 1 | 1 | 0 | 1 | 1 | 13.5 | 75% |
| Rocchi 2004 | 1 | 1 | 0.5 | 0.5 | 1 | 0 | NA | 1 | 0.5 | 0.5 | 0.5 | 0 | 1 | 1 | 1 | 0 | 0 | 1 | 1 | 11.5 | 64% |
| Rocchi 2006 | 1 | 1 | 0 | 0.5 | 1 | 0 | NA | 1 | 1 | 0.5 | 1 | 0 | 1 | 1 | 1 | 0 | 0 | 1 | 1 | 12 | 67% |
| Santos 2017 | 1 | 1 | 1 | 1 | 1 | 1 | 1 | 1 | 1 | 1 | 0.5 | 0 | 0 | 0.5 | 1 | 1 | 1 | 1 | 0.5 | 15.5 | 82% |
| Santos 2017 | 1 | 1 | 1 | 1 | 1 | 1 | 1 | 1 | 1 | 1 | 1 | 0 | 0 | 0 | 1 | NA | 1 | 1 | 0.5 | 14.5 | 81% |
| Santos 2017 | 1 | 1 | 1 | 0.5 | 1 | 0 | 1 | 1 | 1 | 1 | 1 | 0 | 0.5 | 0 | 1 | 0 | 1 | 1 | 0.5 | 13.5 | 71% |
| Sato 2022 | 0.5 | 1 | 1 | 1 | 1 | 1 | 0.5 | 0 | 0.5 | 0 | 1 | 0 | 0 | 0 | 1 | 0 | 1 | 1 | 0.5 | 11 | 58% |
| Schieppati 1994 | 0 | 0 | 0.5 | 0 | 0 | 0 | NA | 0.5 | 1 | 1 | NA | 0 | 0.5 | 1 | 1 | NA | 0 | 1 | 0.5 | 7 | 44% |
| Schieppati 1999 | 1 | 1 | 0.5 | 0.5 | 1 | 0 | NA | 0.5 | 1 | 1 | NA | 0.5 | 0 | 1 | 1 | 1 | 0 | 1 | 0.5 | 11.5 | 68% |
| Schlenstedt 2016 | 1 | 0.5 | 0.5 | 1 | 1 | 0 | NA | 1 | 1 | 1 | 0.5 | 0.5 | 0.5 | 1 | 1 | 1 | 0.5 | 1 | 0.5 | 13.5 | 75% |
| Schmit 2006 | 0.5 | 0.5 | 0.5 | 0.5 | 1 | 0 | NA | 1 | 1 | 1 | 1 | 1 | 0.5 | 0.5 | 1 | 0 | 0 | 1 | 0.5 | 11.5 | 64% |
| Sebastia-Amat 2021 | 1 | 1 | 1 | 1 | 1 | 0 | NA | 0.5 | 1 | 0 | 1 | 0 | 0 | 1 | 1 | 1 | 1 | 1 | 1 | 13.5 | 75% |
| Sebastia-Amat 2023 | 1 | 1 | 1 | 1 | 1 | 1 | NA | 0.5 | 1 | 1 | 1 | 1 | 0 | 1 | 1 | 1 | 1 | 1 | 1 | 16.5 | 92% |
| Severo 2016 | 1 | 1 | 1 | 0.5 | NA | NA | 1 | 0.5 | 0 | 0.5 | 0.5 | 0 | 0 | 0 | 1 | 1 | 1 | 1 | 0.5 | 10.5 | 62% |
| Smart 2023 | 0.5 | 0.5 | 0.5 | 0.5 | 0.5 | 0 | NA | 1 | 1 | 1 | 1 | 1 | 0 | 1 | 1 | 1 | 0.5 | 1 | 0.5 | 12.5 | 69% |
| Sowalsky 2017 | 1 | 1 | 1 | 1 | NA | NA | 1 | 0.5 | 1 | 0 | 0.5 | 0 | 0.5 | 0 | 1 | 1 | 0 | 1 | 1 | 11.5 | 68% |
| Spolaor 2021 | 1 | 1 | 1 | 0.5 | 1 | 0 | 1 | 0.5 | 1 | 0.5 | 0 | 0.5 | 0 | 0 | 1 | 1 | 0.5 | 1 | 0.5 | 12 | 63% |
| Suarez 2011 | 1 | 0.5 | 0.5 | 0.5 | 0 | 0 | NA | 0.5 | 1 | 0.5 | 0 | 1 | 1 | 1 | 1 | 1 | 0 | 1 | 1 | 11.5 | 64% |
| Terra 2020 | 1 | 0.5 | 1 | 1 | 0.5 | 0 | NA | 1 | 1 | 1 | 1 | 0 | 0 | 0.5 | 1 | 0 | 1 | 1 | 1 | 12.5 | 69% |
| Terra 2022 | 1 | 1 | 1 | 1 | 1 | 0 | NA | 0 | 1 | 1 | 1 | 0 | 0 | 0.5 | 1 | 0 | 0.5 | 1 | 1 | 12 | 67% |
| Tollar 2018 | 1 | 1 | 1 | 1 | 1 | 1 | 1 | 0 | 1 | 0 | 0.5 | 0 | 0 | 0 | 1 | NA | 1 | 1 | 0.5 | 12 | 67% |
| Tollar 2018 | 1 | 1 | 1 | 1 | 1 | 1 | 1 | 0 | 1 | 0 | 1 | 0 | 0 | 0 | 1 | 1 | 1 | 1 | 0.5 | 13.5 | 71% |
| Tollar 2019 | 1 | 1 | 1 | 1 | 1 | 1 | 1 | 0 | 1 | 0 | 1 | 0 | 0 | 0 | 1 | 1 | 1 | 1 | 0.5 | 13.5 | 71% |
| Tsai 2022 | 0.5 | 1 | 0.5 | 1 | 1 | 0 | NA | 0 | 1 | 1 | 1 | 0 | 0.5 | 0.5 | 1 | 1 | 1 | 1 | 1 | 13 | 72% |
| Vasconcellos 2023 | 1 | 1 | 1 | 1 | 0.5 | 1 | 1 | 0.5 | 1 | 0 | 1 | 0 | 0 | 1 | 1 | 0 | 1 | 1 | 0.5 | 13.5 | 71% |
| Volpe 2014 | 1 | 1 | 1 | 1 | 1 | 0 | 1 | 0.5 | 1 | 1 | 0 | 0.5 | 0.5 | 0 | 1 | 0 | 1 | 1 | 0.5 | 13 | 68% |
| Wodarski 2023 | 1 | 1 | 0.5 | 1 | 1 | 0.5 | NA | 1 | 1 | 0.5 | 1 | 1 | 1 | 0.5 | 1 | 1 | 0 | 1 | 1 | 15 | 83% |
| Workman 2019 | 1 | 1 | 1 | 0.5 | 1 | 0 | NA | 0.5 | 1 | 0 | 1 | 1 | 1 | 1 | 1 | 1 | 0.5 | 1 | 1 | 14.5 | 81% |
| Yoon 2019 | 0.5 | 1 | 1 | 1 | 1 | 0 | NA | 0.5 | 1 | 0.5 | 0.5 | 0 | 0 | 1 | 1 | 0 | 1 | 1 | 1 | 12 | 67% |
| Yozu 2021 | 1 | 1 | 1 | 0.5 | 1 | NA | NA | 0.5 | 1 | 0.5 | 1 | 1 | 1 | 0 | 1 | NA | 1 | 1 | 1 | 13.5 | 84% |
| Zarucchi 2020 | 1 | 0.5 | 1 | 0.5 | 1 | 0 | 1 | 0 | 1 | 0 | 1 | 1 | 0 | 0 | 1 | NA | 0.5 | 1 | 0.5 | 11 | 61% |
| Zawadka-Kunikowska 2014 | 0.5 | 1 | 0.5 | 1 | 0.5 | 0 | NA | 0 | 1 | 0.5 | 0.5 | 0 | 0 | 0 | 1 | NA | 1 | 1 | 1 | 9.5 | 56% |
| Zawadka-Kunikowska 2022 | 1 | 1 | 0.5 | 1 | 1 | 0 | NA | 0 | 1 | 0.5 | 1 | 1 | 0 | 0 | 1 | 1 | 1 | 1 | 1 | 13 | 72% |
| Zulai 2020 | 1 | 0.5 | 0.5 | 1 | 1 | 0 | NA | 1 | 1 | 0.5 | 0.5 | 0 | 0 | 0 | 1 | 1 | 0 | 1 | 0.5 | 10.5 | 58% |
| Zwergal 2011 | 0.5 | 0.5 | 0.5 | 1 | 1 | 0 | NA | 0.5 | 1 | 0.5 | NA | 0 | 0 | 0 | 0.5 | 1 | 0.5 | 1 | 0.5 | 9 | 53% |
